# Neuroprotective Effects of Human-Induced Pluripotent Stem Cell-Derived Mesenchymal Stem Cell Extracellular Vesicles in Ischemic Stroke Models

**DOI:** 10.3390/biomedicines11092550

**Published:** 2023-09-17

**Authors:** Gang Lu, Xianwei Su, Lihong Wang, Chi-Kwan Leung, Jingye Zhou, Zhiqiang Xiong, Wuming Wang, Hongbin Liu, Wai-Yee Chan

**Affiliations:** 1 CUHK-SDU Joint Laboratory on Reproductive Genetics, School of Biomedical Sciences, The Chinese University of Hong Kong, Hong Kong SAR, China; lugang@cuhk.edu.hk (G.L.); suxianwei@link.cuhk.edu.hk (X.S.); wanglihong1202@163.com (L.W.); leafzhou@link.cuhk.edu.hk (J.Z.); wangwuming@cuhk.edu.hk (W.W.); 2 Center for Neuromusculoskeletal Restorative Medicine, Hong Kong Science Park, Shatin, New Territories, Hong Kong SAR, China; 3 Hong Kong Branch of CAS Center for Excellence in Animal Evolution and Genetics, The Chinese University of Hong Kong, Shatin, New Territories, Hong Kong SAR, China; 4 Key Laboratory for Regenerative Medicine, Ministry of Education, School of Biomedical Sciences, The Chinese University of Hong Kong, Hong Kong SAR, China; 5 Center for Reproductive Medicine, Shandong University, Jinan 250012, China; markxiong@sduivf.com (Z.X.); hongbin_sduivf@aliyun.com (H.L.)

**Keywords:** hiPS-MSC-EV, middle cerebral artery occlusion, proliferation, angiogenesis, exosomes, ischemic stroke

## Abstract

Background: Stroke represents the second leading cause of death and the primary cause of long-term disability in humans. The transplantation of mesenchymal stem cells (MSC) reportedly improves functional outcomes in animal models of cerebral ischemia. Here, we evaluate the neuroprotective potential of extracellular vesicles secreted from human-induced pluripotent stem cell-derived mesenchymal stem cells (hiPS-MSC-EV) using preclinical cell-based and animal-based models of ischemic strokes. Methods: hiPS-MSC-EV were isolated using an ultrafiltration method. HT22 cells were subjected to oxygen-glucose deprivation/reoxygenation (OGD/R) injury for 2 h, followed by treatment with hiPS-MSC-EV (100 μg/mL). Male C57BL/6 mice were subjected to middle cerebral artery occlusion (MCAO) followed by an intravenous injection of hiPS-MSC-EV (100 μg) at three distinct time points. Results: Our experimental approach revealed hiPS-MSC-EV promoted HT22 cell proliferation, reduced apoptosis, and altered cellular morphology following OGD/R. In addition, hiPS-MSC-EV reduced the volume of infarcts, improved spontaneous movement abilities, and enhanced angiogenesis by expressing the VEGF and CXCR4 proteins in the infarcted hemisphere of the MCAO-treated mouse model. Conclusion: Our findings provide evidence of the potential neuroprotective effects of hiPS-MSC-derived extracellular vesicles (hiPS-MSC-EVs) in both in vitro and in vivo mouse models of ischemic stroke. These results suggest that hiPS-MSC-EVs may play a role in neurorestoration and offer insights into potential cell-free strategies for addressing cerebral ischemia.

## 1. Introduction

Stroke represents the second leading cause of death and the primary cause of long-term disability in humans worldwide [1], of which 80% include cases of ischemic stroke [2]. Reperfusion with mechanical thrombectomy and/or thrombolysis is used to restore blood flow in patients with acute stroke episodes but must be administered within 4.5 h after the onset of stroke. Consequently, prehospital time (PHT) is crucial for patients with stroke to improve the stroke management process, as the benefits of thromboflytics are limited to a small proportion of patients [3,4]. Hence, a novel strategy promoting the successful recovery of stroke patients is urgently needed.

In the past two decades, we have witnessed significant advances in understanding the causes and physiology of ischemic stroke. Recently, stem cell-based therapy emerged as a viable neurorestorative therapeutic strategy for stroke patients [5]. In particular, the extracellular vesicles (EVs) secreted by transplanted stem cells are an essential mediator in cellular communication and assist stem cell paracrine action during tissue regeneration [6]. These EVs are very small, nano-sized (50–1000 nm) lipid bilayer spheres that enclose the components of their parent cell, such as RNAs, small non-coding RNAs, and proteins. A diverse array of extracellular vesicles are naturally secreted from a single cell type in vivo [7], whereby the diameter, weight gradient, RNA/protein contents, derived origins, and biological functions of EV subgroups are highly heterogeneous [8].

Extracellular vesicles (EVs) and exosomes are both types of vesicles that are released by cells and play crucial roles in cell-to-cell communication. They carry a variety of biological molecules, such as proteins, lipids, and nucleic acids, which can influence the behavior of recipient cells. They have common properties and correlations. EVs and exosomes can carry proteins, lipids, and nucleic acids from their cell of origin. These can be transferred to recipient cells, altering their behavior. They can influence the behavior of recipient cells and are involved in a range of biological processes, including immune responses, angiogenesis, and cancer. While exosomes are a specific type of EV with unique properties and functions, they share many characteristics with other EVs. Both are important tools for cell-to-cell communication and have potential uses in therapeutics and as biomarkers of disease [9]. Exosomes represent a significant proportion of EV subpopulation types, with a size ranging from 40 to 100 nm in diameter, and generally present surface markers such as CD9, CD81, and CD63 [10]. The isolation of EV subpopulations, such as exosomes, is commonly employed via ultracentrifugation. However, no method efficiently separates extracellular vesicles with high yield and purity. Since EVs can penetrate the blood-brain barrier [8], many studies have focused on the essential properties of extracellular vesicles in regulating normal physiological processes and investigated their role in the pathology of several neurologic diseases [11]. Accordingly, recent studies suggest EVs have the potential to induce neuroprotection and modulate neurite outgrowth and immunosuppression to augment neural repair after stroke, both in vitro and in vivo [12,13,14]. Hence, using MSC secretions, such as exosomes and microvesicles, might offer a viable cell-free therapeutic strategy for treating stroke-related ischemia [12]. MSCs are typically harvested from adult tissues through invasive procedures, and their proliferation and differentiation capacities decrease after several passages in culture. As individuals age, MSC proliferation and differentiation capacity decline significantly [15]. Reprogramming techniques have enabled the generation of induced pluripotent stem cells (iPSCs) from adult somatic cells of patients. It has been proven that iPSCs can be generated from any tissue in the body [16]. These cells possess remarkable growth potential, allowing for the generation of a plentiful source of early-passage MSCs without ethical concerns associated with conventional MSCs [17]. iPSC-derived mesenchymal stem cells (iPSC-MSCs) combine the advantages of both MSCs and iPSCs. Notably, iPSC-MSCs can undergo at least 40 passages in culture while retaining their self-renewal capacity. This set of properties highlights the potential significance of iPSC-MSCs in regenerative medicine and research. [18]. Concerns regarding genotoxicity and tumorigenicity associated with earlier methods of iPSC generation have been substantially reduced through advancements in non-integrating techniques. Reprogramming methods utilizing non-integrating viral vectors (such as Adenoviruses or Sendai viruses) or non-viral vectors (such as episomal vectors, mRNAs, minicircle vectors, and recombinant proteins) eliminate the risk of malignant transformation commonly associated with retroviruses or lentiviruses. Furthermore, these methods reduce the efficiency of repeated transduction, ensuring an appropriate level of transgene expression [19]. There are similarities between iPSC-MSCs and adult MSCs in morphology, surface marker expression profiles, global gene expression, and the capability to differentiate into three lineages [20]. Despite these advantages, concerns about immunological rejection and chromosomal variation persist in cell transplantation therapies [21].

Studies have shown that paracrine activity may contribute to the efficacy of MSC therapy against stroke [22]. Extracellular vesicles (EVs), nanoparticles containing proteins, lipids, nucleic acids, and other biomolecules, play a key role in this process. A beneficial property of EVs is their stability under various physiological conditions and ability to cross the blood-brain barrier (BBB), making them an excellent choice for treating ischemic stroke [23]. EVs derived from allogeneic MSCs, particularly from fetal tissues such as Wharton’s Jelly, placenta, and amniotic fluid, have gained significant attention due to their promising therapeutic potential. These sources offer several advantages, including reduced immunogenicity, an abundance of bioactive molecules, and the potential for off-the-shelf therapy. These EVs can modulate immune responses in acute and chronic inflammatory pathologies by directly targeting the inflammatory microenvironments [24,25]. A previous study showed that bone marrow MSC-derived extracellular vesicles reduced peripheral immunosuppression, enhanced neurovascular regeneration, and improved motor function four weeks after ischemia [26]. Middle cerebral artery occlusion (MCAO) rats achieved better results with intravenous infusion of exosomes in infarct volume, angiogenesis, and alleviated long-term neurological deficits [27]. The application of iPSC-MSC-EVs to enhance angiogenesis in mouse ischemic stroke models has not been reported.

In this study, we isolated hiPS-MSC-EVs and investigated their functional role in neuroprotection using both cell- and animal-based models of ischemic stroke. Our in vitro study demonstrated that hiPSC-MSC-EVs promoted HT22 cell proliferation, reduced apoptosis, and altered cellular morphology. In the MCAO-treated mouse model, hiPSC-MSC-EVs decreased infarcted volume, improved functional outcomes, and enhanced angiogenesis in the ischemic border zone through VEGF and CXCR4 expression. These findings provide the first evidence that hiPSC-MSC-EVs promote neuroprotection in both in vitro and in vivo models of ischemic stroke in mice.

## 2. Methods

### 2.1. Animal Model and Experimental Design

As previously described, we performed an MCAO operation using an intraluminal suture [28]. Wild-type male adult C57BL/6J mice (weight, 25–30 g; 10–12 weeks old) were obtained from the Laboratory Animal Experimental Service Center at the Chinese University of Hong Kong (CUHK). Under anesthesia, the left common carotid artery (LCCA) was carefully dissected free, and a knot was made using 6.0 string, followed by a second knot on the left external carotid artery (LECA). Microvascular clips were applied to the left internal carotid artery (LICA) and the left pterygopalatine artery (LPA). A small incision was made to insert the monofilament into the LICA. After a 60-min occlusion in a heated cage, the monofilament was removed, and a third knot was tied to close the wound. In the case of sham controls, mice were subjected to a similar procedure, except that the filaments did not occlude the MCA and were immediately withdrawn from the internal carotid artery (ICA).

All animal handling and experimental procedures implemented complied with the Chinese University of Hong Kong (CUHK) Schedule 7 Regulations on the Use of Experimental Animals, the International Guiding Principles for Biomedical Research Involving Animals, the Hong Kong Code of Practice for Care and Use of Animals for Experimental Purposes, and the CUHK Guide for Animal Care and Use. The experimental procedures were approved by the University Animal Experiments Ethics Committee of the CUHK (Ref No. 18-121-ITS).

### 2.2. Induction of MSCs from Human iPSCs

Human hiPS cell lines were purchased from Advanced Cell Technology Limited, a company affiliated with Shandong University, and cultured in a mTeSR™1 medium (Cat# 85850. STEMCELL Technology, Vancouver, BC, Canada). iPSCs were cultured in mTeSR1 medium (#85850, Stemcell Technology) on Matrigel-coated vessels (#354277, Corning, New York, NY, USA). For optimal results, cells were grown densely in large colonies. Some cell lines required a single passage adaptation to mTeSR/Matrigel before MSC differentiation. Upon reaching confluence, the medium was switched to an inhibitor differentiation medium known as KOSR + SB (DMEM-Ham’s F-12 basal medium supplemented with 20% knockout serum replacement (KOSR), 1 mM L-glutamine, 10 mM nonessential amino acids, and 10 μM SB431542). This KOSR + SB medium was replaced daily for 10 days. Cells were then harvested using TrypLE Select (#12563011, Gibco, Waltham, MA, USA) to obtain a single-cell suspension. At the start of mesenchymal differentiation, cells were reseeded at 40,000 cells per cm^2^ in the MSC medium. For the next step, cells were replated at 20,000 cells per cm^2^. For subsequent steps, the medium for subculturing cells used MSC medium, which consisted of Dulbecco’s Modified Eagle Medium (Cat# 11885092, Gibco, Waltham, MA, USA) supplemented with 10% FBS, 1% penicillin/streptomycin, 2 mM L-glutamine, and 0.1 mM non-essential amino acids [29]. The cells were successively passaged every 5–7 days until attaining a homogeneous fibroblast morphology and the typical MSC phenotypic characteristics and differentiation potential.

### 2.3. Multipotency of hiPS-MSCs

The functional differentiation capacity of hiPS-MSC into osteogenic, chondrogenic, and adipogenic lineages was tested in a specific medium. For osteogenesis, hiPS-MSC was cultured in osteoblasts (Cat# A1007201, Gibco, USA) for three weeks and then fixed in 4% paraformaldehyde for 30 min at room temperature (RT). The calcification of the matrix was examined using alizarin red staining. Chondrogenesis was studied by culturing hiPS-MSC in a chondrogenic differentiation medium (Cat# A1007101, Gibco, USA) and maintaining it for four weeks in culture. The cells were then fixed in 4% paraformaldehyde at RT for 30 min, and the proteoglycans were detected using Alcian Blue staining. Finally, for adipogenesis differentiation, hiPS-MSC was incubated in an adipogenic medium (Cat# A1007001, Gibco, USA) for two weeks, fixed for 30 min in 4% paraformaldehyde at RT, and the lipid vacuoles were detected using Oil red O staining. The images were captured using a Spot Digital Camera & Leica Microscope Biological Imaging System (Leica, Wetzlar, Germany).

### 2.4. Flow Cytometric Analysis

Flow cytometry was used to characterize the surface markers of MSC as previously described [30]. After 4–5 passages, the cells were harvested using trypsin-EDTA (Invitrogen, Waltham, MA, USA) and incubated with 3% bovine serum albumin (BSA, Gibco, USA) in PBS for 30 min to block non-specific antigen binding. The cells were incubated with primary antibodies from the human MSC analysis kit (Cat#562245, BD Biosciences, San Jose, CA, USA) to identify the characteristic human MSC surface markers. The following antibodies were used: APC-conjugated anti-CD73, PerCP-conjugated anti-CD105, and fluorescein isothiocyanate (FITC)-conjugated anti-CD90. Additionally, negative cell surface markers were examined using PE hMSC negative cocktail antibody solution, including PE CD45, PE CD34, PE CD11b, PE CD19, and PE HLA-DR. To detect non-specific fluorescence, isotype-matched mouse monoclonal antibodies were utilized. The surface antigens were analyzed using a BD LSR Fortessa cell analyzer (BD Biosciences, USA). Since the exosomes are too small to be detected using flow cytometry, binding them first to the antibody-coated beads is necessary. According to the manufacturer’s instructions, isolated exosomes were purified using the Exosome-Human Isolation/Detection Reagent. The titrated exosomes were mixed with anti-CD9 coating magnetic beads (Cat#10620D, Invitrogen, USA) or anti-CD81 coating magnetic beads (Cat#10622D, Invitrogen, USA) and incubated overnight (18–22 h) at 2 °C to 8 °C with sample mixing. The bead-bound exosomes were then collected by immunomagnetic cell separation and centrifugation and stained with anti-CD63-PE (Cat# 12-0639-41, Invitrogen, USA) for 60 min at room temperature. The isotypes were used for each experiment as negative controls. The samples were analyzed using a BD LSR Fortessa cell analyzer (BD Biosciences, USA).

### 2.5. Exosome Isolation and Identification

Exosomes were collected using differential centrifugation as previously described [31]. Briefly, the cells were grown to 80% confluency and then cultured in a chemically defined exosome-free medium for 48 h. This medium consisted of 10% exosome-free FBS (#A2720801, Gibco, USA) in the MSC medium. The conditioned medium (CM) was obtained and centrifuged sequentially at 300× *g* for 10 min and 2000 g for 10 min at 4 °C. To remove cellular debris, the supernatant was filtered through a 0.22-μm filter (Millipore, Burlington, MA, USA). After this, the supernatant was centrifuged at 4000× *g* at 4 °C to reduce the volume to ~200 μL by ultra-filtration in a 15-mL Amicon Ultra-15 Centrifugal Filter Unit (Millipore, 100 KD). Finally, the 200 μL centrifuged liquid was diluted in 15 mL of PBS to remove the residual medium, and the previous centrifugation and ultra-filtration steps were repeated. The total protein concentration in the exosomes was determined using the bicinchoninic acid (BCA) assay (Thermo Fisher, Waltham, MA, USA), as previously described [32].

Exosome morphology was visualized on a Hitachi HT-7700 transmission electron microscope (Hitachi, Japan), and the images were captured using a digital camera (Olympus, Japan). In brief, freshly isolated exosomes were placed on a copper grid coated with 0.125% Formvar in chloroform. The grids were stained with 1% (*v*/*v*) uranyl acetate in ddH2O, and exosome samples were examined immediately. Alternatively, the isolated exosomes could be fixed with cold 2.5% *v*/*v* glutaraldehyde in 0.1 M PBS, rinsed in PBS, dehydrated using a graded series of ethanol, and embedded in Spurr resin. Ultrathin sections (60 nm) were stained with uranyl acetate and lead citrate. Imaging was performed using a Hitachi H7700 Transmission Electron Microscope. Antibodies against CD9 (1:200; sc-13118, Santa Cruz, CA, USA), CD63 (1:200; ab216130, Abcam, Cambridge, UK), and CD81 (1:100; sc-166029, Santa Cruz), Calnexin (1:200; sc-23954, SantaCruz), Tubulin (#15115, Cell Signaling Technology, Danvers, MA, USA) were used to target the exosomes. Exosome RNA was extracted using the TRIzol-LS reagent. The exosome was resuspended in 200 μL PBS, and then TRIzol lysis reagent was mixed with the exosome solution. Chloroform was added, thoroughly mixed, and centrifuged at 12,000× *g* for 15 min. The aqueous phase was mixed with isopropanol and purified using a spin column. Finally, RNA was eluted in 30 μL of nuclease-free water. RNA concentration was assessed using a NanoDrop 2000 spectrophotometer (Thermo Scientific, Waltham, MA, USA). The RNA yield and size distribution were analyzed using an Agilent 2100 Bioanalyzer with an RNA 6000 Pico kit (Agilent Technologies, Foster City, CA, USA). As part of evaluating the EVs’ characteristics, nanoparticle tracking analysis (NTA) was executed using a Flow NanoAnalyzer (NanoFCM N30E, Xiamen, China). Briefly, A cocktail of silica nanospheres (68, 91, 113, and 151 nm, provided by NanoFCM) and 200 nm polystyrene beads were used to calibrate the instrument for concentration and size. Particle events were recorded for one minute after EVs preparations were diluted (typically 1:150 dilution for 10 K fractions and 1:200 dilution for EVs). An intensity matrix, a calibration curve, and a flow rate were used to calculate particle numbers.

### 2.6. OGD Model

Oxygen glucose deprivation (OGD) injury models were used to mimic stroke, as previously described [33]. HT22 murine hippocampal cells were seeded into a 100 cm^2^ dish and maintained at 37 ℃/5% CO_2_ in an incubator. We used DMEM high glucose supplemented with 10% FBS as the standard medium. Before the OGD treatment, the culture medium was replaced with normal DMEM plus 10% exosome-free FBS (#A2720801, Gibco, USA). For the OGD treatment, the culture medium was changed to a deoxygenated glucose-free DMEM without FBS. The cells were then placed in a hypoxia chamber (Billups-Rothenberg, San Diego, CA, USA), flushed with 95% N_2_ and 5% CO_2_ for 10 min, and sealed for the OGD treatment. HT22 cells were exposed to the gas mixture in the hypoxic box for 2 h. After the deprivation period, the cells were changed to a medium containing exosome-free FBS, without FBS, or with the EVs and re-oxygenated for 24 h.

### 2.7. Cell Proliferation and Apoptosis

HT22 cells were seeded into 96-well culture plates at a density of 1 × 10^3^ cells/well in 100 μL of DMEM supplemented with 10% FBS. After 24 h culture, the cells were treated with OGD/R. Cell viability was examined at the end of the reoxygenation period using the MTT assay (MTT, Thermo Fisher Scientific, Waltham, MA, USA) according to the manufacturer’s instructions. After removing the medium, formazan was solubilized by adding 50 μL of dimethylsulfoxide (DMSO) followed by 10μL MTT solution (5 mg/mL in PBS) to each well, after which the cells were incubated for 4 h at 37 ℃. The absorbance at 540 nm was measured using a microplate spectrophotometer (Bio-Rad Lab, Berkeley, CA, USA). The relative cell viability was calculated using the OD540 value for the OGD group as a proportion of the mean OD540 value for the control group. The experiment was repeated at least three times.

The cells were collected, and the apoptosis rate was assessed using the Dead Cell Apoptosis Kit (Cat#V13241, Invitrogen, USA) with Annexin V and Propidium Iodide (PI). Briefly, the cells were collected with a cell scraper after reoxygenation, washed in cold phosphate-buffered saline (PBS), and resuspended in annexin binding buffer, to which we added 5 μL of Alexa Fluor^®^ 488 Annexin V and 1 μL 100 μg/mL PI to each 100 μL of cell suspension. This mixture was incubated for 15 min, and finally analyzed using the BD LSR Fortessa cell analyzer (BD Biosciences, USA). The FITC was represented by the FL1 channel, PI staining was represented by the FL2 channel, and the cells stained with Annexin-V and PI were considered the apoptotic cell population.

### 2.8. RNA Sequencing and Analysis

RNA sequencing (RNA-seq) was performed by Novogene (Beijing, China). Nine samples were divided into three groups, one of which was the control group, while the others represented the OGD and OGE groups (GEO accession number GSE208008). RNA extraction was performed using TRIZOL reagent (Invitrogen, USA) according to the manufacturer’s instructions. RNA integrity and quantity (IQ) were determined by the Bioanalyzer 2100 system (Agilent, Santa Clara, CA, USA). Next, Illumina NovaSeq 6000 was used to sequence RNA libraries using a 150 bp end pairing protocol. Approximately 70 million clean reads per sample were generated from raw data after removing adapter reads, N base reads, and low-quality reads. Clean reads were aligned to the reference genome using HISAT2 (v2.0.5). Q20, Q30, and GC contents of clean reads were also calculated. Each gene’s read count was calculated using FeatureCounts (v1.5.0-p3). Differential expression gene (DEG) analysis was performed in R (v3.3.2) using the DESeq2 v1.15.28 package. To improve the accuracy of DEGs, we defined genes with a fold change ≥1.5 and adjusted *p*-value ≤ 0.05 as significant DEGs. The Ingenuity Pathway Analysis (IPA) (www.ingenuity.com/products/ipa, accessed on 19 November 2020) software was also performed to predict upstream regulators with their activation score and *p*-value. The IPA software analyzed the top network, disease, and disorder enrichment.

### 2.9. Neurologic Function Analysis

Neurological tests were performed on days 0, 3, 14, and 28 after MCAO (*n* = 6 mice/group). We used the modified neurological severity score (NSS) and Zea Longa to evaluate the functional results as described previously [28]. The Zea longa scoring method was used, assigning a score from 0–4 based on the level of neural damage. Scores between 1 and 3 indicated successful modeling. A score of 0 indicated no impairment in neural function, while a score of 4 indicated acute damage. The battery included tests for nerve motor function, gait, balance, and reflexes for the NSS test. In motor function tests, mice were lifted by their tail, allowing free movement. Limb buckling and head movement on the vertical axis were recorded over 30 s. Gait analysis involved placing the mouse on a flat surface and observing its walking; the inability to walk straight, circling, or falling to the side of paralysis was recorded. To assess balance function, mice were placed on a circular beam, noting whether they could grasp or embrace the beam, the number of limbs falling from the beam, and the time until they fell. For the reflex test, head vibrations when the auditory canal was touched indicated positive auricle reflexes and blinking when gently touching the cornea with cotton indicated positive corneal reflexes. The score was graded from 0 to 14 (where 0 is normal and 14 shows the largest defect). Each evaluation was repeated in triplicate, and the average score was recorded.

### 2.10. Histological Analysis

Hematoxylin and Eosin (H&E) staining were used to detect the lesion volume. In animals stained with H&E, the brains of MCAO and sham mice were removed similarly and stored in 10% buffered formalin acetate for at least 24 h at 4 ℃. After fixation, the brains were embedded in paraffin and sectioned into 5-μm slices. We selected 100 μm intervals to be stained with H&E. The sections were imaged under a light microscope and digitized with a charge-coupled device camera Spot Digital Camera & Leica Microscope Biological Imaging System (Leica, Germany). H&E staining exhibited a pale coloration in the areas of ischemic lesions. We used the following formula to calculate the lesion volume as a percentage of the volume of the contralateral hemisphere: % lesion volume = (volume of the contralateral hemisphere-ipsilateral intact volume)/volume of contralateral hemisphere × 100.

Slides that were formalin-fixed and embedded in paraffin were deparaffinized using ethanol and xylene. Antigen retrieval was performed, followed by an overnight incubation at 4 °C using the following primary antibodies: anti-VEGF (ab51745, Abcam), SDF-1α mAb (sc-518066, Santa Cruz), and CXCR4 (ab181020, Abcam). The antigen-antibody reactions were visualized using a DAB Substrate Kit (ab64238, Abcam). The slides were counterstained with hematoxylin, dehydrated, and mounted in the final steps.

### 2.11. Western Blot Analysis

The brain tissues of mice were obtained from the infarcted hemisphere on day 28 after MCAO. The tissue extracts were dissociated by RIPA buffer. Total protein concentration was quantified using a BCA kit (Bio-Rad Lab, CA, USA). Equal protein lysates were loaded to 10% sodium dodecyl SDS-PAGE and transferred onto polyvinylidene difluoride membranes (Bio-Rad Lab, CA, USA). After blocking with 5% non-fat milk, the membranes were incubated with primary antibodies against CXCR4, VEGF, and SDF-1α overnight at 4 ℃. The membranes were then incubated with horseradish peroxidase-conjugated secondary antibody at a 1:2000 dilution (Cell Signaling Technology, USA). Immunoreactive proteins were then exposed to X-ray film by enhanced chemiluminescence.

### 2.12. Statistical Analysis

All data were expressed as the mean ± SD. The GraphPad Prism 8 software was used to analyze the data. Parametric tests were used for data that met normality and equal variance assumptions. A student’s *t*-test was used to analyze the differences between two unpaired samples. A two-way analysis of variance (ANOVA) followed by post hoc analysis was used for multiple comparisons. *p* < 0.05 was used as the statistically significant threshold.

## 3. Results

### 3.1. Successful Induction of hiPSs into hiPS-MSCs

**Figure 1:**
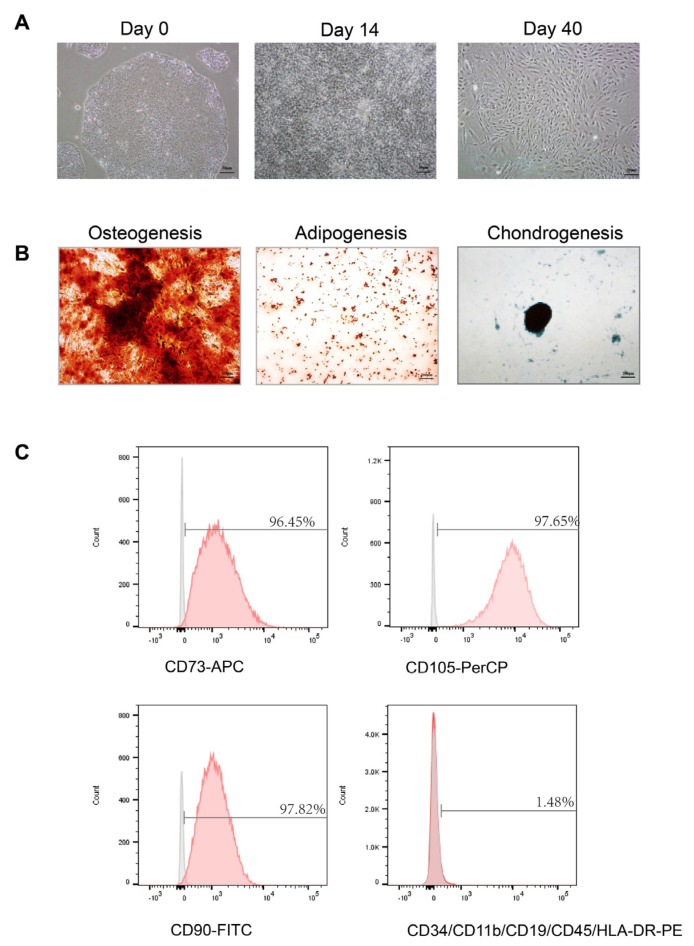
Characterization of human-induced pluripotent stem cell-derived mesenchymal stem cells (hiPS−MSCs) (**A**). Phase-contrast image of hiPS before differentiation (Day 0); Intermediate phase of MSCs differentiation from hiPS (Day 14); Typical morphology of MSC cells (Day 40). Original magnification 200×, Scale bar, 50 μm; (**B**). Multi-lineage differentiation of hiPS−MSCs. Alizarin Red, Alcian blue, and Oil red staining were used for osteogenesis, chondrogenesis, and adipogenesis. Original magnification 40×, Scale bar, 200 μm; (**C**). Flow cytometry analysis of the positive surface markers CD73, CD90, CD105, and negative surface markers CD34, CD45, CD11b, CD19 and HLA-DR in hiPS-MSCs.

**Figure 2:**
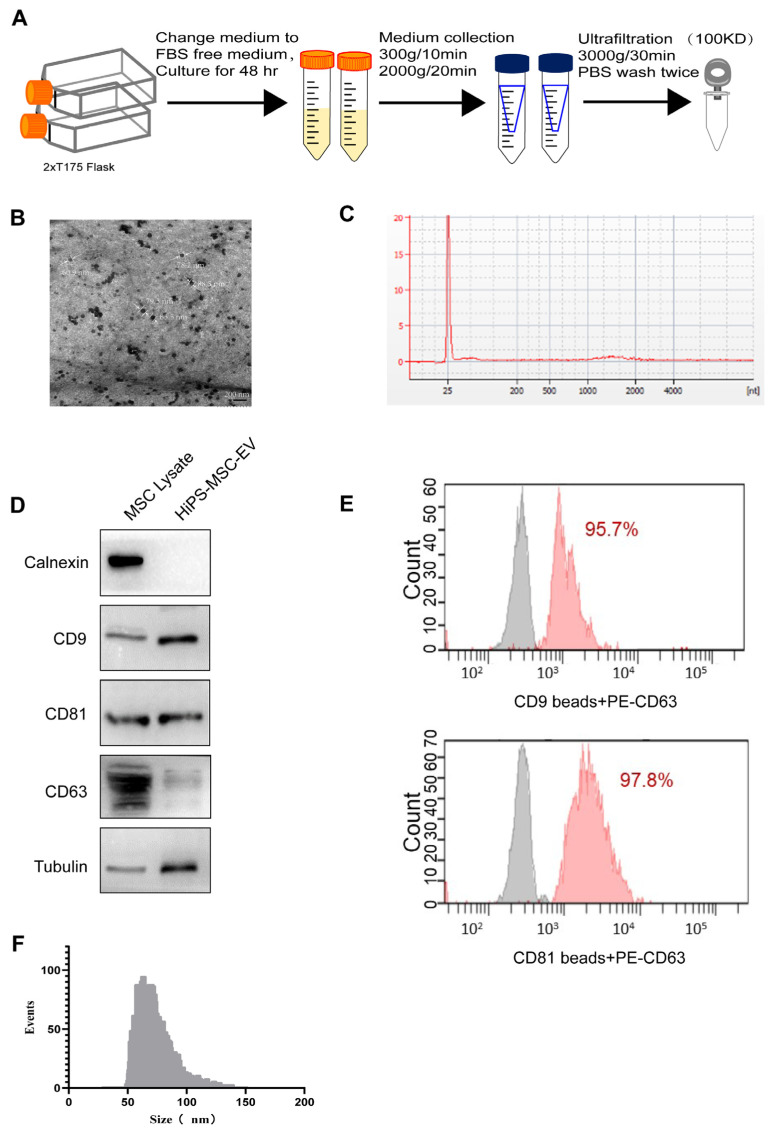
Characterization of hiPS-MSCs derived extracellular vesicles (hiPS-MSCs-EV) (**A**). Flowchart of hiPS-MSC-EVs isolation from the culture medium. (**B**). Morphology of hiPS-MSC-EVs under transmission electron microscopy. (**C**). Representative bioanalyzer profile of the RNA contained in hiPS-MSC-EVs. (**D**). Western blotting analysis of exosomal positive markers CD63, CD9, and CD81 and negative marker Calnexin in MSC lysate and hiPS-MSC-EVs. Tubulin was used as the loading control. (**E**). Flow cytometry analysis of positive marker CD63 expression after binding to CD9 and CD81 antibody-coated beads. The solid gray line represents cells that were unstained. while solid pink line represents the experimental samples. (**F**). Particle size distribution of iPSC-MSC-EVs measured using a flow nanoanalyzer.

MSC was successfully induced from hiPS using a one-step induction protocol. We observed a tendency for cells to form packed clones with a high nucleus/cytoplasm ratio during incubation. On day 14, the hiPSs exhibited a monolayer with a more prominent spindle-shaped morphology at the border of the colonies. On day 40, hiPS-MSCs displayed a homogeneous fibroblast-like morphology after several passages (Figure 1A). Tri-lineage MSC differentiation experiments examined the multipotency property of hiPS-MSCs; the hiPS-MSCs were cultured in the appropriate induction media suitable for osteogenesis, adipogenesis, and chondrogenesis differentiation. Osteogenic differentiation was highly efficient as more than 80% of cells were stained with Alizarin Red after three weeks of induction. Adipogenic differentiation was also highly efficient, with more than 90% of cells showing positive staining with Oil Red O. Finally, Alcian blue staining suggested efficient chondrogenic differentiation, with more than 70% of cells showing positive staining in both hiPS-MSCs types (Figure 2B).

Specific surface antigen profiling of hiPS-MSCs was measured using fluorescence-activated cell sorting (FACS). A multicolor analysis panel was performed to detect ISCT-recommended surface markers. HiPS-MSCs exhibited more than 95% of the population with the ISCT-defined positive expression markers, including CD73, CD90, and CD105. The negatively expressed markers (CD45/CD34/CD11b/CD19/HLA-DR PE) were detected in less than 2% of the population in pretreated cocktails (Figure 1C).

### 3.2. Characterization of hiPS-MSC-EVs

Ultrafiltration was employed for extracellular vesicles (EVs) from the hiPS-MSCs cell culture medium, as shown in the flowchart of Figure 2A. A transmission electron microscope (TEM) revealed a cup- or round-shaped morphology in hiPS-MSC-EVs with sizes ranging from 50 to 100 nm (Figure 2B). RNA was extracted from EVs using the miRCURY RNA isolation reagent, which can extract RNA of all sizes, including small RNA molecules. The size of exosomal RNAs was examined with an Agilent 2100 Bioanalyzer. Our results showed peaks of small RNA (25–200 nucleotides) in exosomal RNAs (Figure 2C). Western blotting confirmed the expression of the specific markers CD9, CD63, and CD81 in hiPS-MSC-EVs.

In contrast, the negative marker calnexin, an endoplasmic reticulum marker, was not detected in hiPS-MSC-EVs (Figure 2D). To better characterize the subpopulation of hiPS-MSC-EVs, we performed FACS to detect the expression of surface markers. The results indicated high ratios of hiPS-MSC-EVs expressing CD63 after binding to CD9 (95.7%) and CD81 antibody-coated beads (97.8%) (Figure 2E). Flow nanoanalyzer analysis revealed the iPSC-MSC-EVs were mostly distributed between 50 and 100 nm in diameter, with an average diameter of 70.25 nm (Figure 2F).

### 3.3. hiPS-MSC-EVs Inhibited Apoptosis and Promoted Cell Proliferation in OGD-Treated HT22 Cells

**Figure 3:**
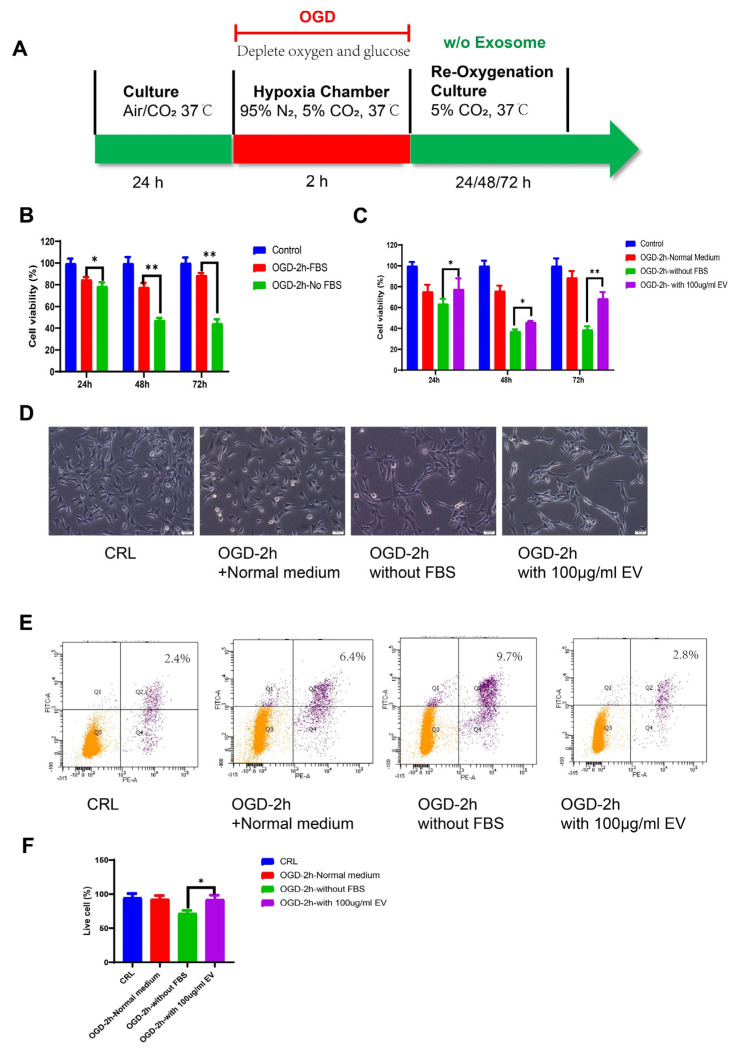
The effects of hiPS-MSC-EVs on cellular proliferation and apoptosis in HT22 cell lines from the mouse hippocampus. (**A**). Flowchart of OGD model and hiPS-MSC-EVs treatment. (**B**). HT22 cells were detected using the MTT assay after OGD treatment at 24 h, 48 h, and 72 h (*n* = 6 per group). (**C**). HT22 cells were detected using the MTT assay after OGD treatment and were further treated with hiPS-MSC-EVs for 24 h (*n* = 6 per group). (**D**). The morphology of cells treated with/without hiPS-MSC-EVs was examined under a microscope. Original magnification 200×, Scale bar, 50 μm; (**E**). HT22 cells treated with/without hiPS-MSC-EVs were stained with Annexin/PI, and apoptosis was examined by flow cytometry. (**F**). The experiment was repeated three times. The proportion of living cells in each group is also shown (*n* = 3 per group). Student *t* test. In all panels, data were expressed as means ± SD, * *p* < 0.05, ** p<0.01.

We next evaluated the effect of hiPS-MSC-EVs on cell viability and proliferation using a model of neuronal HT22 cells with oxygen-glucose deprivation/reoxygenation (OGD/R) in vitro (Figure 3A). To establish the OGD model, the cells were incubated in a hypoxia chamber for different periods, including 10 min, 20 min, 30 min, 1 h, 2 h, and 6 h. After OGD treatment, the cells were re-oxygenated in a 5% CO_2_ cell culture chamber for 24 h and examined by microscopy and proliferation assays. The results showed that the more prolonged OGD treatments (i.e., 6 h) led to dramatic apoptotic morphology and that proliferation decreased after re-oxygenation. The cells could not be recovered after the OGD treatment. In the 2 h OGD treatment, a significant decrease of absorbance at 490 nm (i.e., cell density) and minor morphology change was observed (Figure S1A). At the end of the reoxygenation period, cell viability was examined using the MTT assay at different times, specifically 24 h, 48 h, and 72 h. Our analysis revealed that the viability of HT22 cells was markedly decreased in OGD/NO FBS compared to the positive control OGD/FBS group, which indicated the cellular ischemic model was successfully established (Figure 3B, statistical test in Table S1).

To evaluate the effects of hiPS-MSC-EVs in the OGD model, HT22 cells were rescued with/without EV products after 2 h of OGD treatment. Cell viability and proliferation were again examined using the MTT assay. Moreover, the HT22 cells were treated with different EV doses, i.e., 20, 50, and 100 μg/mL, which revealed a dose-dependent effect on the successful rescue of HT22 cells after 24 h OGD treatment (Figure S1B). Cell proliferation was then examined at different time points (i.e., 24 h, 48 h, and 72 h). It showed that the hiPS-MSC-EVs treatment (100 μg/mL) exhibited significant proliferation compared to the control group without EV product treatment (Figure 3C, statistical test in Table S2). The morphology of the cells treated with/without hiPS-MSC-EVs was examined under a microscope. It unveiled a dramatic apoptotic morphology in cells treated with hiPS-MSC-EVs (100 μg/mL) compared to the control group (no EV product treatment) at 24 h (Figure 3D). Flow cytometry examined the living and apoptotic cells after OGD and reoxygenation with/without hiPS-MSC-EVs. The results showed that hiPS-MSC-EVs significantly enhanced cell viability after OGD treatment compared to the PBS-treated control group, with the proportion of apoptotic cells decreasing from 9.7 to 2.8% (Figure 3E). Finally, the number of living cells was calculated and repeated three times (Figure 3F, statistical test in Table S3).

### 3.4. Transcriptome Analysis Revealed the Expression Profile and Potential Pathways Involved in Ischemic Stroke in Mice

**Figure 4:**
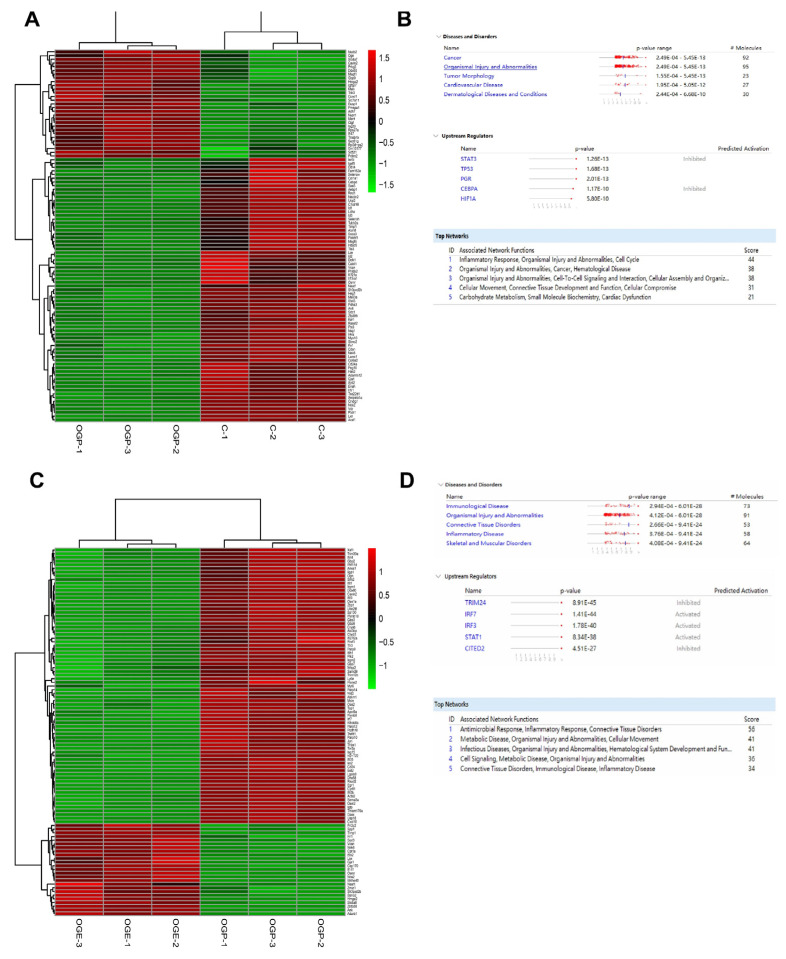
Transcriptome analysis of hiPS −MSC−Evs in HT22 cell lines from the mouse hippocampus. (**A**). Heatmap and hierarchical clustering analysis of RNA-seq data for the differentially expressed genes (DEGs). Columns in the heatmap represent OGP and control samples (*n* = 3 per group); rows indicate individual genes. (**B**). IPA core analysis of the comparison dataset from OGP and control groups. (**C**). Columns in the heatmap represent OGE and OGP samples; rows indicate individual genes. (**D**). IPA core analysis of the comparison dataset from OGE and OGP groups. Control group: HT22 cells without treatment; OGP group: HT22 cells with OGD and reoxygenation with PBS; OGE: HT22 cells with OGD and reoxygenation with hiPS−MSC−EVs.

We evaluated differentially expressed genes (DEGs) using a heatmap and hierarchical clustering of RNA-seq data. The heatmap revealed the expression patterns of the top 100 DEGs of the samples clustered according to biological replicates within the OGD (*n* = 3) and control (*n* = 3) groups (Figure 4A). The comparison dataset from the two groups was submitted to the IPA tool for core analysis to allow further understanding of the biological functions and pathways involved and to test whether the DEGs genes are associated with the regulation of ischemia. Several diseases and disorders were identified with this approach, including cancer, organismal injury and abnormalities, tumor morphology, cardiovascular disease, and dermatological diseases associated with the upstream regulators STAT3, TP53, PGR, CEBPA, and HIF1A. The top 5 associated networks were also ranked based on this dataset (Figure 4B). The heatmap displayed the expression profile of 100 DEGs by comparing the OGD and OGE groups (Figure 4C).

We used the IPA tool and again identified several diseases and disorders, including immunological disease, organismal injury and abnormalities, connective tissue disorders, inflammatory disease, and skeletal and muscular disorders, associated with the upstream regulators *Trim24*, *Irf7*, *Irf3*, *Stat1*, and *Cited2*. The top 5 associated networks were also ranked based on this dataset (Figure 4D). These results indicate that several genes and their associated regulators play an essential role in the pathology of ischemia.

### 3.5. hiPS-MSC-EVs Improve Neurologic Outcome and Lesion Volume

**Figure 5:**
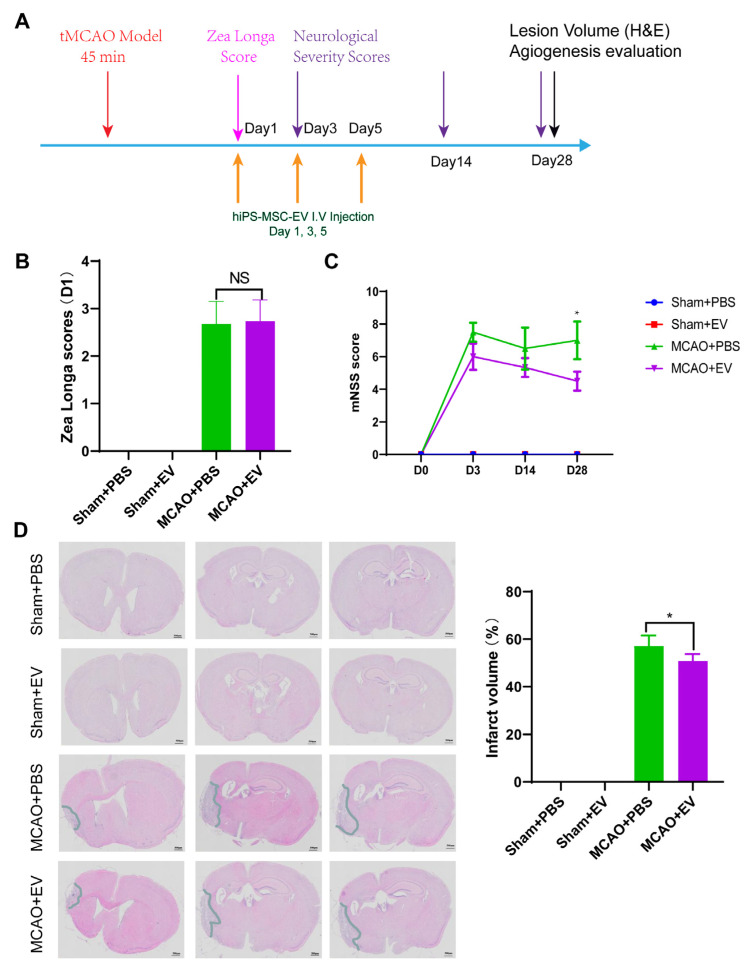
hiPS-MSC-EVs influence ischemic stroke volume and animal behavior in the mouse model of middle cerebral artery occlusion (MCAO). (**A**). Schematic representation of the establishment of the MCAO model and hiPS-MSC-EVs treatment. (**B**). Zea longa score evaluation. Two-way ANOVA test (*n* = 4 per group). (**C**). mNSS score evaluation. Two-way ANOVA test (*n* = 4 per group). (**D**). The lesion volume included all injury-related changes visible using H&E staining. Column displayed the quantification of lesion volume (*n* = 5 per group). Original magnification 20×, Scale bar, 500 μm; Student *t* test. In all panels, data were expressed as means ± SD, NS represents no significance, * *p* < 0.05.

After administering hiPS-MSC-EVs to MCAO mice, we assessed the neurological outcome, lesion volume (infarct size), and angiogenesis at different time points (Figure 5A). Behavioral deficits were evaluated using two neurological function tests, the Zea–Longa score and the modified neurological severity scores (mNSS). A higher score on each test represents a more severe behavioral deficit. The Zea–Longa scores obtained in the MCAO group were significantly higher than those calculated in the sham group, with no significant changes observed between the sham control and the Sham+EV groups (Figure 5B, statistical test in Table S4) (*n* = 4 per group). However, the mNSS score in the MCAO+EV group gradually decreased after surgery. As early as three days after MCAO, the hiPS-MSC-EVs treatment effects for the functional outcomes were small on days 3 and 14. However, significant changes were observed at day 28 post-treatment (Figure 5C, statistical test in Table S5). These results suggest the hiPS-MSC-EVs improve neurological function on day three after MCAO compared to the MCAO-only group (*n* = 4 per group).

Moreover, 28 days after the onset of stroke, the ischemic lesion volumes of the two groups were 57.08% + 4.48% (MCAO+PBS) and 50.76% + 2.91% (MCAO+EV), whereby a decrease occurred in the latter. No significant differences in lesion volume were observed between the sham+PBS and sham+EV treatment groups (Figure 5D, statistical test in Table S6) (*n* = 4 per group). These results indicate that the neurological function of MCAO mice can be restored by hiPS-MSC-EVs treatment.

### 3.6. hiPS-MSC-EVs Promote the Expression of VEGF and CXCR4 in the Ischemic Region

**Figure 6:**
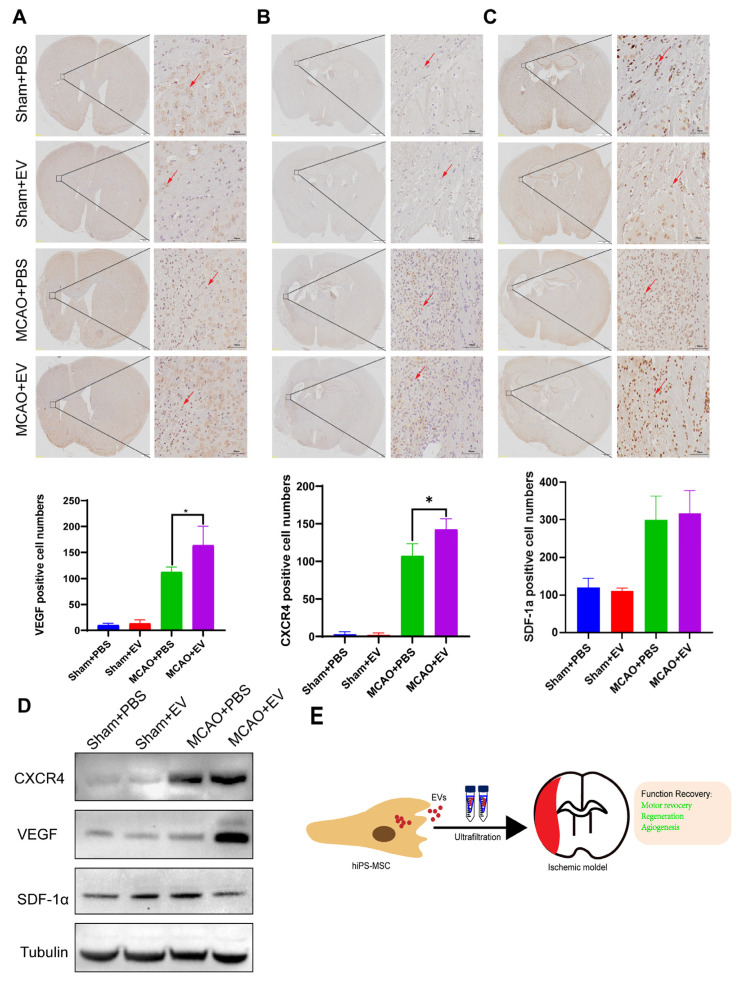
hiPS-MSC-EVs promote angiogenesis in the infarcted hemisphere. (**A**). Immunohistochemical analysis of VEGF in the infarcted hemisphere and quantification analysis of VEGF were performed (*n* = 6 per group). Positively stained cells are brown (indicated by the red arrow). (**B**). Immunohistochemical analysis of CXCR4 in the infarcted hemisphere and quantification analysis of CXCR4 were performed (*n* = 6 per group). Positively stained cells are brown (indicated by the red arrow). (**C**). Immunohistochemical analysis of SDF-1 in infarcted hemisphere. Quantification analysis of SDF-1 was performed (*n* = 6 per group). Positively stained cells are brown (indicated by the red arrow). (**D**). The expression of SDF-1, CXCR4, and VEGF was determined by western blot analysis. (**E**). Schematic representation of hiPS-MSC-EVs function in the ischemic mice model. Original magnification 20×, Scale bar, 500 μm; Inset magnification 200×, Scale bar, 50 μm. In all panels, data were expressed as means ± SD, * *p* < 0.05).

The expression of VEGF, SDF-1α, and CXCR4 in the infarcted hemisphere was evaluated by immunohistochemistry (IHC) staining. Our results showed that VEGFA was significantly increased in the MCAO+EV group compared to the MCAO+PBS group (Figure 6A), with CXCR4 following a similar but less pronounced trend (Figure 6B). No differences were found in the case of SDF-1α (Figure 6C) (statistical test in Tables S7–S9, *n* = 6 per group). The protein levels of the three genes were detected by western blot analysis and confirmed VEGF and CXCR4 expression were significantly increased in the MCAO+EV compared to the MCAO+PBS group and in both groups compared to the sham group (Figure 6D). Our results suggest that hiPS-MSC-EVs can inhibit hippocampal cell apoptosis and promote cell proliferation in the cellular model. The ischemic stroke performed in the MCAO mouse model exhibited function recovery, including motor recovery, regeneration, and angiogenesis (Figure 6E).

## 4. Discussion

Despite recent improvements in ischemic stroke patient care and neurorehabilitation, the long-term disabilities associated with stroke remain a significant problem in medicine, whereby devising effective therapeutic interventions to improve neuronal recovery in stroke patients is essential. The study showed that hiPS-MSC-EV improved HT22 cell proliferation, attenuated apoptosis, and altered cellular morphology after OGD/R treatment vitro. hiPS-MSC-EVs reduce the lesion volume and improve spontaneous movement ability and neurological deficits, as shown in the two neurological scores (Zea Longa and mNSS scores). Moreover, hiPS-MSC-EVs enhance angiogenesis in vivo by expressing VEGF and CXCR4 in the ischemic border zone of MCAO-treated mice.

Stem cell transplantation is emerging as a promising treatment for ischemic diseases, as research has confirmed the beneficial therapeutic effects of stem cells in both clinical and experimental models [34]. Among various stem cells, adult mesenchymal stem cells (MSCs), particularly bone marrow-derived stem cells (BMSCs), show potential due to their high self-renewal, differentiation capacity, and low immunogenicity for the treatment of ischemic diseases [35,36]. Limitations include restricted available BMSCs from patients and an age-related decline in their proliferation and differentiation potential. To address these challenges, induced pluripotent stem cells (iPSCs) have been explored. They can be derived from patients’ somatic cells, eliminating ethical issues and immune rejection, iPSC-derived MSCs have shown superior proliferation capacity compared to primary BMSCs. MSCs pose the risk of genetic and epigenic alterations [37]. Stem cells are now believed to function not only via cell replacement but also through a paracrine effect, releasing growth factors, cytokines, chemokines, and extracellular vesicles (EVs). These components aid cell regeneration or angiogenesis. EVs secreted by stem cells have attracted attention in the ‘paracrine hypothesis’ due to their unique characteristics and positive effects in treating ischemic injury [38,39]. MSC-derived EVs may mitigate risks associated with direct MSC transplantation, such as uncontrolled differentiation, immune rejection, or tumorigenicity while maintaining regenerative and immunomodulatory properties. Therefore, they present a viable therapeutic alternative. Emerging evidence consistently showed that exosomes derived from stem cells have promising therapeutic potential in various preclinical disease models. Exosome-based cell-free therapy is expected to overcome current limitations and challenges associated with cell-based therapy, such as the ease of passing through the BBB in the ischemic brain due to their lipophilicity, lower risks of mounting graft versus host immune response and tumorigenicity and occlusion in the microvasculature [40,41,42].

Over 70% of infarcts occur in the MCA and its branches [43]. Currently, preclinical models studying the pathophysiology and therapeutic intervention in ischemic stroke are under development and active investigation [44]. MCAO is a commonly used method to occlude ECA to reduce cerebral blood flow in murine models without craniotomy [45]. The MCAO model, characterized by highly reproducible infarcts [46], is useful for reproducing and studying ischemic stroke and its associated cell death, inflammation, and damaging effects on BBB [47]. However, other cerebral ischemic models, such as photothrombotic and thromboembolic 4-VO and 3-VO, ventricular fibrillation, or neck tourniquet ischemia models use different animals (e.g., gerbil) and did not examine the functional role of the hiPS-MSC-EV under different ischemia-inducing protocols in vitro and in vivo.

It was recently reported that the intravenous administration of human umbilical cord-derived MSCs into Sprague Dawley Rats attenuates ischemic brain damage with a significant reduction of infarct size and swelling [40]. However, the mNSS scores between the exosome-treated and control groups did not differ significantly different. In addition, the study also showed that exosome treatment attenuated somatosensory functional recovery and motor functions as measured by the mean beam walking score and latency to fall [40]. Accordingly, these previous results are consistent with another study that showed the mNSS scores of exosomes derived from the bone marrow of adult male Wistar rats and vehicle-treated groups did not differ after seven days of reperfusion [13]. Neuroprotective effects of exosomes released by MSCs and other cell types, such as astrocytes and brain endothelial cells, have also been observed following stroke in MSCs and other cell types [48]. Exosomes administered intravenously to MCAO rats improved foot faults and modified neurologic severity scores compared to PBS-treated rats [13].

Additionally, adipose-derived MSCs (ADMSCs) exosome therapy could reduce brain infarction zones and enhance neurological recovery. The similarities between the findings from this study and prior research provide validation for our results [49]. The discrepancy in the post-stroke outcome after the administration of stem cell-derived exosomes could be explained by the analysis of different species, treatment protocols, the origin of stem cells, or distinct dosing regimens [13,26,40].

It has been postulated that the microenvironments exposed to stem cells play a crucial role in the protection mechanisms exerted by stem cells and the secreted exosome. MSCs derived from human umbilical cord blood induced apoptosis in cancerous cells but suppressed apoptosis in spinal cord-injured rat models [50,51,52]. The expression levels of several miRNAs, such as *miR-133b* released from MSCs, originated from different sources in rats and drastically differed upon exposure to normal brain tissues or ischemic brain extracts [14]. The characterization of exosomes derived from stem cells cultured in different microenvironments is thus of significant biological and clinical relevance to determine their clinical efficacy in post-stroke neurological recovery.

While our study provides promising insights into the neuroprotective potential of hiPS-MSC-EVs in ischemic stroke, it is important to acknowledge several limitations. Firstly, the small sample size in our study might limit our findings’ statistical power and generalizability. Furthermore, the exclusive use of male C57BL/6 mice may not fully account for gender-specific physiological differences, thereby limiting the extrapolation of our results to female subjects. Additionally, the use of a mouse model raises concerns about the direct translatability of our findings to human biology, considering inherent species differences. Moreover, our in vitro model, utilizing HT22 cells subjected to a 2-h OGD/R injury, may oversimplify the complexities of real-world ischemic brain environments. While we observed enhanced angiogenesis through increased VEGF and CXCR4 protein expression, the multifaceted nature of angiogenesis warrants incorporating a broader spectrum of markers for a comprehensive understanding. Lastly, the lack of a specified extended observation period post-treatment prevents the exploration of potential long-term effects or side effects associated with hiPS-MSC-EVs. Despite these limitations, our findings lay the groundwork for more extensive studies that can further investigate the therapeutic potential of hiPS-MSC-EVs in ischemic stroke.

To test functional conservation, it is thus necessary to conduct further investigations into the functional specificities of hiPS-MSC-EVs in different hippocampal or neuronal cell lines originating from different organisms. In addition, the functional specificity of other downstream pathways associated with ischemia identified here using the IPA analysis and RNA-seq data warrant additional work to decipher the specific mechanisms mediating neuroprotection. A future comprehensive study of the inflammatory response and activation of other apoptotic pathways during cerebral ischemic injury is paramount to understanding the cellular responses to ischemia and the actions of hiPS-MSC-EVs in mediating neuroprotection in vitro and in vivo.

## 5. Conclusions

This study suggested hiPS-MSC-EVs inhibit apoptosis and promote cell proliferation using the OGD/R-injury HT22 cell model and enhance neurological recoveries, such as motor recovery, regeneration, and angiogenesis, in the MCAO-treated mice model. This work advances the current understanding of the functional role of hiPS-MSC-EVs. It supports the development of a novel therapeutic strategy against ischemic injury to improve post-stroke neurological function.

## Data Availability

All data generated or analyzed in this study are included in this published article.

## Supplementary Materials

The following supporting information can be downloaded at: https://www.mdpi.com/article/10.3390/biomedicines11092550/s1, Figure S1: The effect of hiPS-MSC-EVs on the HT22 cellular OGD model; Table S1: Comparison of Cell viability (%) within OGD treatment. Student t test results shown; Table S2: Comparison of Cell viability (%) within OGD and EVs treatment. Student t test results shown; Table S3:Comparison of Live cell (%) within OGD and EVs treatment. Student t test results shown; Table S4: Comparison of Zea Longa score within MCAO and MCAO+EV treatment. Student t test results shown; Table S5: Comparison of mNSS score within MCAO and MCAO+EV treatment. Two-way ANVOA test results shown; Table S6: Comparison of infarct volume (%) within MCAO and MCAO+EV treatment. Student t test results shown; Table S7: VEGF positive cell numbers within MCAO and MCAO+EV treatment. Student t test results shown; Table S8: CXCR4 positive cell numbers within MCAO and MCAO+EV treatment. Student t test results shown; Table S9: SDF-1a positive cell numbers within MCAO and MCAO+EV treatment. Student t test results shown.

## References

[1] Feigin V.L., Abajobir A.A., Abate K.H., Abd-Allah F., Abdulle A.M., Abera S.F., Abyu G.Y., Ahmed M.B., Aichour A.N., Aichour I. (2017). Global, regional, and national burden of neurological disorders during 1990–2015: A systematic analysis for the global burden of disease study 2015. Lancet Neurol..

[2] Chen M., Lyu H., Li T., Su X.W., Leung C.K., Xiong M.Z.Q., Poon W.S., Cai Y.F., Lu G., Chan W.Y. (2020). Study of the association between gait variability and gene expressions in a mouse model of transient focal ischemic stroke. Int. J. Neurosci..

[3] Soto-Camara R., Gonzalez-Bernal J., Aguilar-Parra J.M., Trigueros R., Lopez-Liria R., Gonzalez-Santos J. (2021). Factors related to prehospital time in caring for patients with stroke. Emergencias.

[4] Powers W.J., Derdeyn C.P., Biller J., Coffey C.S., Hoh B.L., Jauch E.C., Johnston K.C., Johnston S.C., Khalessi A.A., Kidwell C.S. (2015). 2015 american heart association/american stroke association focused update of the 2013 guidelines for the early management of patients with acute ischemic stroke regarding endovascular treatment a guideline for healthcare professionals from the american heart association/american stroke association. Stroke.

[5] Andres R.H., Horie N., Slikker W., Keren-Gill H., Zhan K., Sun G.H., Manley N.C., Pereira M.P., Sheikh L.A., McMillan E.L. (2011). Human neural stem cells enhance structural plasticity and axonal transport in the ischaemic brain. Brain.

[6] Sun B.C., Peng J., Wang S.F., Liu X.J., Zhang K.H., Zhang Z.Z., Wang C., Jing X.G., Zhou C.F., Wang Y. (2018). Applications of stem cell-derived exosomes in tissue engineering and neurological diseases. Rev. Neurosci..

[7] Margolis L., Sadovsky Y. (2019). The biology of extracellular vesicles: The known unknowns. PLoS Biol..

[8] Raposo G., Stoorvogel W. (2013). Extracellular vesicles: Exosomes, microvesicles, and friends. J. Cell Biol..

[9] Colombo M., Raposo G., Thery C. (2014). Biogenesis, secretion, and intercellular interactions of exosomes and other extracellular vesicles. Annu. Rev. Cell Dev. Biol..

[10] Crescitelli R., Lässer C., Szabó T.G., Kittel A., Eldh M., Dianzani I., Buzás E.I., Lötvall J. (2013). Distinct rna profiles in subpopulations of extracellular vesicles: Apoptotic bodies, microvesicles and exosomes. J. Extracell. Vesicles.

[11] Lee Y., El Andaloussi S., Wood M.J. (2012). Exosomes and microvesicles: Extracellular vesicles for genetic information transfer and gene therapy. Hum. Mol. Genet..

[12] Xin H., Li Y., Liu Z., Wang X., Shang X., Cui Y., Zhang Z.G., Chopp M. (2013). Mir-133b promotes neural plasticity and functional recovery after treatment of stroke with multipotent mesenchymal stromal cells in rats via transfer of exosome-enriched extracellular particles. Stem. Cells.

[13] Xin H., Li Y., Cui Y., Yang J.J., Zhang Z.G., Chopp M. (2013). Systemic administration of exosomes released from mesenchymal stromal cells promote functional recovery and neurovascular plasticity after stroke in rats. J. Cereb Blood Flow. Metab..

[14] Xin H., Li Y., Buller B., Katakowski M., Zhang Y., Wang X., Shang X., Zhang Z.G., Chopp M. (2012). Exosome-mediated transfer of mir-133b from multipotent mesenchymal stromal cells to neural cells contributes to neurite outgrowth. Stem. Cells.

[15] Zaim M., Karaman S., Cetin G., Isik S. (2012). Donor age and long-term culture affect differentiation and proliferation of human bone marrow mesenchymal stem cells. Ann. Hematol..

[16] Takahashi K., Yamanaka S. (2006). Induction of pluripotent stem cells from mouse embryonic and adult fibroblast cultures by defined factors. Cell.

[17] Hu G.W., Li Q., Niu X., Hu B., Liu J., Zhou S.M., Guo S.C., Lang H.L., Zhang C.Q., Wang Y. (2015). Exosomes secreted by human-induced pluripotent stem cell-derived mesenchymal stem cells attenuate limb ischemia by promoting angiogenesis in mice. Stem. Cell Res. Ther..

[18] Lian Q., Zhang Y., Zhang J., Zhang H.K., Wu X., Zhang Y., Lam F.F., Kang S., Xia J.C., Lai W.H. (2010). Functional mesenchymal stem cells derived from human induced pluripotent stem cells attenuate limb ischemia in mice. Circulation.

[19] Thanaskody K., Jusop A.S., Tye G.J., Wan Kamarul Zaman W.S., Dass S.A., Nordin F. (2022). Mscs vs. Ipscs: Potential in therapeutic applications. Front. Cell Dev. Biol..

[20] Hynes K., Menicanin D., Han J., Marino V., Mrozik K., Gronthos S., Bartold P.M. (2013). Mesenchymal stem cells from ips cells facilitate periodontal regeneration. J. Dent. Res..

[21] Okano H., Nakamura M., Yoshida K., Okada Y., Tsuji O., Nori S., Ikeda E., Yamanaka S., Miura K. (2013). Steps toward safe cell therapy using induced pluripotent stem cells. Circ. Res..

[22] Park W.S., Ahn S.Y., Sung S.I., Ahn J.Y., Chang Y.S. (2018). Strategies to enhance paracrine potency of transplanted mesenchymal stem cells in intractable neonatal disorders. Pediatr. Res..

[23] Zhang Z.G., Buller B., Chopp M. (2019). Exosomes-beyond stem cells for restorative therapy in stroke and neurological injury. Nat. Rev. Neurol..

[24] Cargnoni A., Papait A., Masserdotti A., Pasotti A., Stefani F.R., Silini A.R., Parolini O. (2021). Extracellular vesicles from perinatal cells for anti-inflammatory therapy. Front. Bioeng. Biotechnol..

[25] Drobiova H., Sindhu S., Ahmad R., Haddad D., Al-Mulla F., Al Madhoun A. (2023). Wharton’s jelly mesenchymal stem cells: A concise review of their secretome and prospective clinical applications. Front. Cell Dev. Biol..

[26] Doeppner T.R., Herz J., Gorgens A., Schlechter J., Ludwig A.K., Radtke S., de Miroschedji K., Horn P.A., Giebel B., Hermann D.M. (2015). Extracellular vesicles improve post-stroke neuroregeneration and prevent postischemic immunosuppression. Stem. Cells Transl. Med..

[27] Xia Y., Ling X., Hu G., Zhu Q., Zhang J., Li Q., Zhao B., Wang Y., Deng Z. (2020). Small extracellular vesicles secreted by human ipsc-derived msc enhance angiogenesis through inhibiting stat3-dependent autophagy in ischemic stroke. Stem. Cell Res. Ther..

[28] Wang J., Liu X., Lu H., Jiang C., Cui X., Yu L., Fu X., Li Q., Wang J. (2015). Cxcr4(+)cd45(−) bmmnc subpopulation is superior to unfractionated bmmncs for protection after ischemic stroke in mice. Brain Behav. Immun..

[29] Chen Y.S., Pelekanos R.A., Ellis R.L., Horne R., Wolvetang E.J., Fisk N.M. (2012). Small molecule mesengenic induction of human induced pluripotent stem cells to generate mesenchymal stem/stromal cells. Stem Cells Transl. Med..

[30] Zhang J., Guan J., Niu X., Hu G., Guo S., Li Q., Xie Z., Zhang C., Wang Y. (2015). Exosomes released from human induced pluripotent stem cells-derived mscs facilitate cutaneous wound healing by promoting collagen synthesis and angiogenesis. J. Transl. Med..

[31] Nong K., Wang W., Niu X., Hu B., Ma C., Bai Y., Wu B., Wang Y., Ai K. (2016). Hepatoprotective effect of exosomes from human-induced pluripotent stem cell-derived mesenchymal stromal cells against hepatic ischemia-reperfusion injury in rats. Cytotherapy.

[32] Oh M., Lee J., Kim Y.J., Rhee W.J., Park J.H. (2018). Exosomes derived from human induced pluripotent stem cells ameliorate the aging of skin fibroblasts. Int. J. Mol. Sci..

[33] Guo F., Wang H., Li L., Zhou H., Wei H., Jin W., Wang Q., Xiong L. (2013). A novel domain of amino-nogo-a protects ht22 cells exposed to oxygen glucose deprivation by inhibiting nadph oxidase activity. Cell Mol. Neurobiol..

[34] Van Nguyen T.T., Vu N.B., Van Pham P. (2021). Mesenchymal stem cell transplantation for ischemic diseases: Mechanisms and challenges. Tissue Eng. Regen. Med..

[35] Kim S.G., George N.P., Hwang J.S., Park S., Kim M.O., Lee S.H., Lee G. (2023). Human bone marrow-derived mesenchymal stem cell applications in neurodegenerative disease treatment and integrated omics analysis for successful stem cell therapy. Bioengineering.

[36] Guo Y., Yu Y., Hu S., Chen Y., Shen Z. (2020). The therapeutic potential of mesenchymal stem cells for cardiovascular diseases. Cell Death Dis..

[37] Neri S. (2019). Genetic stability of mesenchymal stromal cells for regenerative medicine applications: A fundamental biosafety aspect. Int. J. Mol. Sci..

[38] Gnecchi M., Zhang Z., Ni A., Dzau V.J. (2008). Paracrine mechanisms in adult stem cell signaling and therapy. Circ. Res..

[39] Keshtkar S., Azarpira N., Ghahremani M.H. (2018). Mesenchymal stem cell-derived extracellular vesicles: Novel frontiers in regenerative medicine. Stem Cell Res. Ther..

[40] Nalamolu K.R., Venkatesh I., Mohandass A., Klopfenstein J.D., Pinson D.M., Wang D.Z., Veeravalli K.K. (2019). Exosomes treatment mitigates ischemic brain damage but does not improve post-stroke neurological outcome. Cell Physiol. Biochem..

[41] Dasari V.R., Spomar D.G., Gondi C.S., Sloffer C.A., Saving K.L., Gujrati M., Rao J.S., Dinh D.H. (2007). Axonal remyelination by cord blood stem cells after spinal cord injury. J. Neurotrauma.

[42] Kenmuir C.L., Wechsler L.R. (2017). Update on cell therapy for stroke. Stroke Vasc. Neurol..

[43] Bogousslavsky J., Van Melle G., Regli F. (1988). The lausanne stroke registry: Analysis of 1000 consecutive patients with first stroke. Stroke.

[44] Dias L.A.A., Colli B.O., Netto J.C., Lachat J.J. (2000). Focal cerebral ischaemia induced by middle cerebral artery occlusion and the neuroprotective effect of ketoprofen in rats. Arq. Neuro-Psiquiat..

[45] Fluri F., Schuhmann M.K., Kleinschnitz C. (2015). Animal models of ischemic stroke and their application in clinical research. Drug Des. Dev. Ther..

[46] Liu S., Zhen G., Meloni B.P., Campbell K., Winn H.R. (2009). Rodent stroke model guidelines for preclinical stroke trials (1st edition). J. Exp. Stroke Transl. Med..

[47] Howells D.W., Porritt M.J., Rewell S.S., O’Collins V., Sena E.S., van der Worp H.B., Traystman R.J., Macleod M.R. (2010). Different strokes for different folks: The rich diversity of animal models of focal cerebral ischemia. J. Cereb. Blood Flow Metab..

[48] Venkat P., Cui C., Chopp M., Zacharek A., Wang F., Landschoot-Ward J., Shen Y., Chen J. (2019). Mir-126 mediates brain endothelial cell exosome treatment-induced neurorestorative effects after stroke in type 2 diabetes mellitus mice. Stroke.

[49] Chen K.H., Chen C.H., Wallace C.G., Yuen C.M., Kao G.S., Chen Y.L., Shao P.L., Chen Y.L., Chai H.T., Lin K.C. (2016). Intravenous administration of xenogenic adipose-derived mesenchymal stem cells (admsc) and admsc-derived exosomes markedly reduced brain infarct volume and preserved neurological function in rat after acute ischemic stroke. Oncotarget.

[50] Chelluboina B., Klopfenstein J.D., Pinson D.M., Wang D.Z., Veeravalli K.K. (2014). Stem cell treatment after cerebral ischemia regulates the gene expression of apoptotic molecules. Neurochem. Res..

[51] Gondi C.S., Veeravalli K.K., Gorantla B., Dinh D.H., Fassett D., Klopfenstein J.D., Gujrati M., Rao J.S. (2010). Human umbilical cord blood stem cells show pdgf-d-dependent glioma cell tropism in vitro and in vivo. Neuro Oncol..

[52] Dasari V.R., Veeravalli K.K., Tsung A.J., Gondi C.S., Gujrati M., Dinh D.H., Rao J.S. (2009). Neuronal apoptosis is inhibited by cord blood stem cells after spinal cord injury. J. Neurotrauma.

