# Supplementary material for: Neuroprotective Effects of Human-Induced Pluripotent Stem Cell-Derived Mesenchymal Stem Cell Extracellular Vesicles in Ischemic Stroke Models
Source: Biomedicines. 2023 Sep 17;11(9):2550. doi: 10.3390/biomedicines11092550 (PMC10525838; doi:10.3390/biomedicines11092550)
Supplement: Supplementary file 1 [file biomedicines-11-02550-s001.zip › Table S1-S9.pdf]

Supplementary Table S1. Comparison of Cell viability (%) within OGD treatment. Student t test results shown.

**Group**	**Mean**	**S.D.**	**N**	**P value**	**Comparison variables**
Control-24hr	99.89	4.26	6	NA	NA
OGD-2hr-FBS-24hr	84.89	2.3	6	1.87137E-05	OGD-2hr-FBS-24hr VS Control-24hr
OGD-2hr-No FBS-24hr	78.88	3.43	6	0.005106764	OGD-2hr-No FBS-24hr VS OGD-2hr-FBS
Control-48hr	99.84	5.76	6	NA	NA
OGD-2hr-FBS-48hr	77.92	3.75	6	1.44575E-05	OGD-2hr-FBS-48hr VS Control-48hr
OGD-2hr-No FBS-48hr	47.45	2.06	6	8.18525E-09	OGD-2hr-No FBS-48hr VS OGD-2hr-FBS-48hr
Control-72hr	100.14	4.92	6	NA	NA
OGD-2hr-FBS-72hr	88.94	2.05	6	0.01488723	OGD-2hr-FBS-72hr VS Control-72hr
OGD-2hr-No FBS-72hr	44.70	3.55	6	4.80457E-05	OGD-2hr-No FBS-72hr VS OGD-2hr-FBS-72hr

Supplementary Table S2. Comparison of Cell viability (%) within OGD and EVs treatment. Student t test results shown.

**Group**	**Mean**	**S.D.**	**N**	**P value**	**Comparison variables**
Control-24hr	100.08	3.65	6	NA	NA
OGD-2hr-Normal medium-24hr	75.52	6.34	6	0.00012093	OGD-2hr-Normal medium-24hr VS Control-24hr
OGD-2hr-without FBS-24hr	63.74	4.55	6	0.008567348	OGD-2hr-without FBS-24hr VS OGD-2hr-Normal medium-24hr
OGD-2hr-with 100 µg/ml EV-24hr	77.83	10.19	6	0.022423269	OGD-2hr-with 100 µg/ml EV-24hr VS OGD-2hr-without FBS-24hr

Control-48hr	100.05	4.95	6	NA	NA
OGD-2hr-Normal medium-48hr	75.99	5.01	6	2.24651E-05	OGD-2hr-Normal medium-48hr VS Control-48hr
OGD-2hr-without FBS-48hr	37.28	1.81	6	2.07635E-07	OGD-2hr-without FBS-48hr VS OGD-2hr-Normal medium-48hr
OGD-2hr-with 100 μg/ml EV-48hr	46.11	0.95	6	5.09341E-05	OGD-2hr-with 100 μg/ml EV-48hr VS OGD-2hr-without FBS-48hr
Control-48hr	100	7.1	6	NA	NA
OGD-2hr-Normal medium-48hr	88.88	6.15	6	0.015834995	OGD-2hr-Normal medium-72hr VS Control-72hr
OGD-2hr-without FBS-48hr	38.93	3.07	6	5.08876E-08	OGD-2hr-without FBS-72hr VS OGD-2hr-Normal medium-72hr
OGD-2hr-with 100 μg/ml EV-48hr	68.72	6.09	6	1.01304E-05	OGD-2hr-with 100 μg/ml EV-72hr VS OGD-2hr-without FBS-72hr

Supplementary Table S3. Comparison of Live cell (%) within OGD and EVs treatment. Student t test results shown.

**Group**	**Mean**	**S.D.**	**N**	**P value**	**Comparison variables**
Control	95	6	3	NA	NA
OGD-2hr-Normal medium	93	5	3	0.6814	OGD-2hr-Normal medium VS Control
OGD-2hr-without FBS	72	4	3	0.0052	OGD-2hr-without FBS VS OGD-2hr-Normal medium
OGD-2hr-with 100 μg/ml EV	92	6	3	0.0085	OGD-2hr-with 100 μg/ml EV VS OGD-2hr-without FBS

Supplementary Table S4. Comparison of Zea Longa score within MCAO and MCAO+EV treatment.

Student t test results shown.

**Group**	**Mean**	**S.D.**	**N**	**P value**	**Comparison variables**
Sham+PBS	0	0	4	NA	NA
Sham+EV	0	0	4	NA	NA
MCAO+PBS	3	0.82	4	0.0052	MCAO+PBS VS Sham+EV
MCAO+EV	3.25	0.5	4	0.6376	MCAO+EV VS MCAO+PBS

Supplementary Table S5. Comparison of mNSS score within MCAO and MCAO+EV treatment.

Two-way ANVOA test results shown.

**Group**	**Mean**	**S.D.**	**N**	**Source of Variation (% of total variation)**
Sham+PBS-D0	0	0	4	Interaction 2.417<br>P=0.0295<br>Row Factor 86.76<br>P<0.0001<br>Column Factor 4.862<br>P<0.0001
Sham+EV-D0	0	0	4	
MCAO+PBS-D0	0	0	4	
MCAO+EV-D0	0	0	4	
Sham+PBS-D3	0	0	4	
Sham+EV-D3	0	0	4	
MCAO+PBS-D3	7.5	0.58	4	
MCAO+EV-D3	6	0.82	4	
Sham+PBS-D14	0	0	4	
Sham+EV-D14	0	0	4	
MCAO+PBS-D14	6.5	1.30	4	
MCAO+EV-D14	5.3	0.58	4	
Sham+PBS-D28	0	0	4	
Sham+EV-D28	0	0	4	
MCAO+PBS-D28	7	1.15	4	
MCAO+EV-D28	4.5	0.58	4	

Supplementary Table S6. Comparison of infarct volume (%) within MCAO and MCAO+EV treatment. Student t test results shown.

Group	Mean	S.D.	N	P value	Comparison variables
Sham+PBS	0	0	4	NA	NA
Sham+EV	0	0	4	NA	NA
MCAO+PBS	57.08	4.48	4	<0.0001	MCAO+PBS VS Sham+EV
MCAO+EV	50.76	2.91	4	0.0296	MCAO+EV VS MCAO+PBS

Supplementary Table S7. VEGF positive cell numbers within MCAO and MCAO+EV treatment. Student t test results shown.

Group	Mean	S.D.	N	P value	Comparison variables
Sham+PBS	10.33	3.27	6	NA	NA
Sham+EV	13.33	7.04	6	0.350618753	Sham+EV VS Sham+PBS
MCAO+PBS	112.20	9.5	6	3.85917E-11	MCAO+PBS VS Sham+EV
MCAO+EV	165.83	36.56	6	0.013846922	MCAO+EV VS MCAO+PBS

Supplementary Table S8. CXCR4 positive cell numbers within MCAO and MCAO+EV treatment. Student t test results shown.

Group	Mean	S.D.	N	P value	Comparison variables
Sham+PBS	2.83	3.37	6	NA	NA
Sham+EV	1.88	2.57	6	0.64497438	Sham+EV VS Sham+PBS
MCAO+PBS	107	16.37	6	1.68366E-09	MCAO+PBS VS Sham+EV
MCAO+EV	142.33	14.29	6	0.048026665	MCAO+EV VS MCAO+PBS

Supplementary Table S9. SDF-1a positive cell numbers within MCAO and MCAO+EV treatment. Student t test results shown.

**Group**	**Mean**	**S.D.**	**N**	**P value**	**Comparison variables**
Sham+PBS	120.44	24.47	6	NA	NA
Sham+EV	110.67	8.19	6	0.272348184	Sham+EV VS Sham+PBS
MCAO+PBS	299.5	63.94	6	2.55893E-07	MCAO+PBS VS Sham+EV
MCAO+EV	317.75	60.47	6	0.566852842	MCAO+EV VS MCAO+PBS
