# Supplementary figures and images for: Neuroprotective Effects of Human-Induced Pluripotent Stem Cell-Derived Mesenchymal Stem Cell Extracellular Vesicles in Ischemic Stroke Models
Source: Biomedicines. 2023 Sep 17;11(9):2550. doi: 10.3390/biomedicines11092550 (PMC10525838; doi:10.3390/biomedicines11092550)

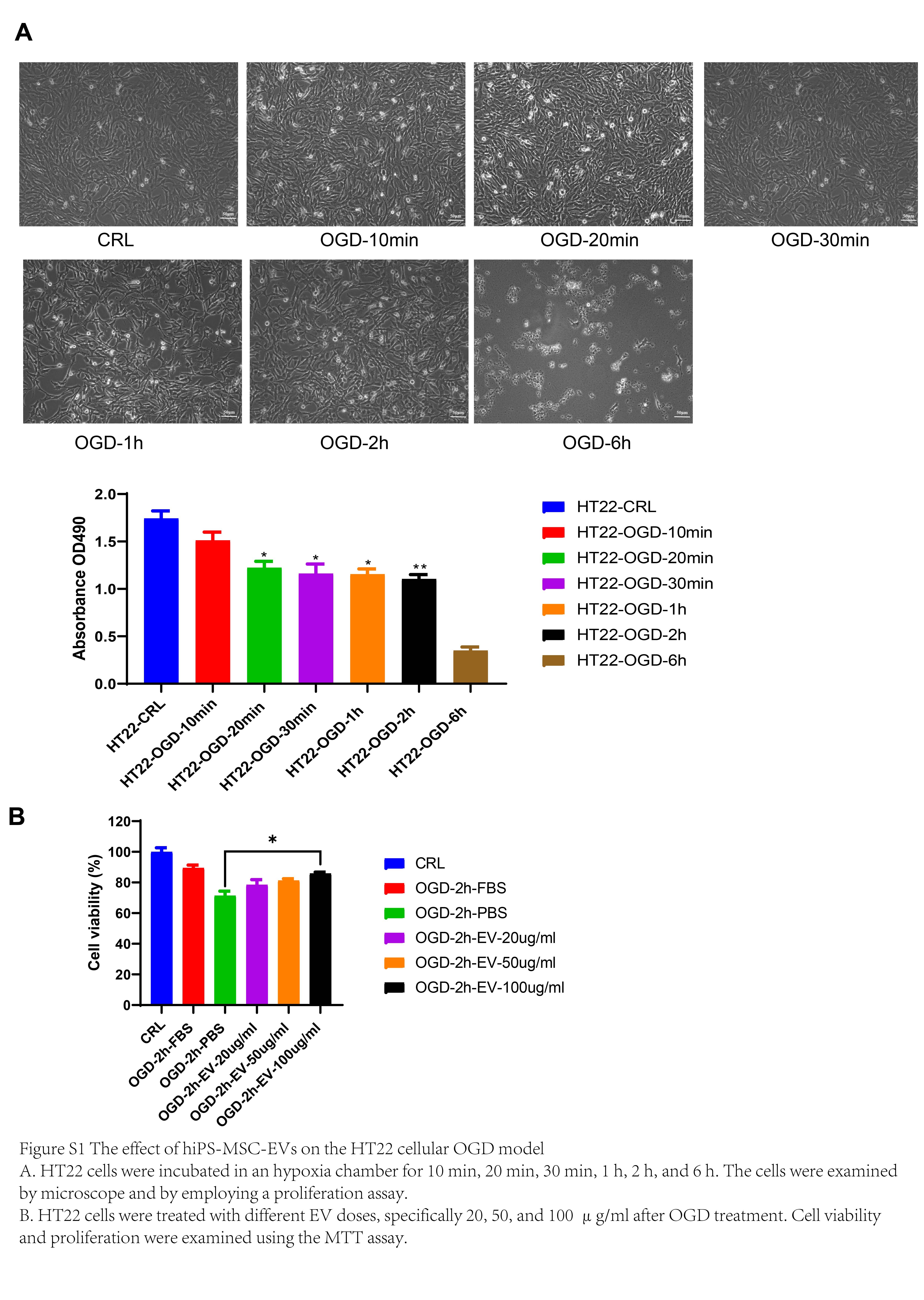

Supplement: Supplementary file 1 [file biomedicines-11-02550-s001.zip › Figure S1.jpg]
